# Editorial: Parochial Altruism: Pitfalls and Prospects

**DOI:** 10.3389/fpsyg.2016.01004

**Published:** 2016-06-30

**Authors:** Hannes Rusch, Robert Böhm, Benedikt Herrmann

**Affiliations:** ^1^Experimental and Applied Psychology, Faculty of Behavioral and Movement Sciences, Vrije Universiteit AmsterdamAmsterdam, Netherlands; ^2^Peter Löscher Chair of Business Ethics, TUM School of Management, Technische Universität MünchenMunich, Germany; ^3^Decision Analysis, School of Business and Economics, RWTH Aachen UniversityAachen, Germany; ^4^School of Economics, Faculty of Social Sciences, University of NottinghamNottingham, UK

**Keywords:** parochial altruism, intergroup conflict, in-group favoritism, intergroup relations, evolution

A number of recent publications have promoted the idea that the high levels of pro-sociality and violent intergroup conflict observed in humans might result from a joint evolution of behavioral traits causing cooperativeness and altruism among members of the same group (“in-group love”) and spite and aggression between different groups (“out-group hate”). This hypothesis, dating back to Darwin (1871), has been dubbed “parochial altruism” (Choi and Bowles, 2007; also see: de Dreu et al., 2014; Rusch, 2014a; Yamagishi and Mifune, 2016).

Research on group conditional pro- and anti-social behaviors has a long tradition in psychology (see e.g., Tajfel, 1982; Yamagishi and Mifune, 2009). By suggesting an evolutionary link between “in-group love” and “out-group hate,” though, parochial altruism theory sparked renewed interdisciplinary interest in this topic (e.g., Bernhard et al., 2006; de Dreu et al., 2010; García and van den Bergh, 2011; Abbink et al., 2012; Ockenfels and Werner, 2014).

Darwin's idea that more cooperative groups had better survival chances throughout our species' supposedly very violent (pre)history (Bowles, 2009), and that in-group directed altruism and out-group directed hostility could have evolved together seems intuitively plausible. In fact, Choi and Bowles (2007) have shown that it is logically consistent, given that a number of assumptions about the frequency, brutality, and strategic structure of ancestral intergroup conflicts hold. Evidence for the correctness of these assumptions is mixed, though (Fry and Söderberg, 2013; Rusch, 2014a; Yamagishi and Mifune, 2016). Therefore, a series of recent papers have argued that these assumptions need to be refined (Rusch, 2013, 2014b; Weisel and Böhm, 2015; Böhm et al., 2016).

The ten original studies included in this Research Topic investigate selected assumptions and predictions of parochial altruism theory in detail. We, the editors, are convinced that their highly instructive findings will help researchers interested in parochial altruism, but also in intergroup psychology more generally, to gain a much more fine-grained understanding of the interplay of altruistic and spiteful motives in human decision making in the context of intergroup relations.

The broad range of disciplines represented by the authors contributing to this Research Topic and the variety of methods used in their studies are representative for the current interdisciplinary interest in parochial altruism. The most important insight that, in our view, can be derived from the works collected here is that human decision making in intergroup contexts is more complex than suggested by current theory. Thus, we hope that future theorizing on parochial altruism will be stimulated by the evidence gathered in this Research Topic (also see Everett et al. for suggestions of future research directions). In the remainder of this editorial, we briefly highlight central findings reported here, which, to us, appear most informative for prospective enhancements of parochial altruism theory.

To our knowledge, Cacault et al. provide some of the first evidence of “unprovoked” parochial altruism in a laboratory setting. Using an iterated asymmetric variant of the Intergroup Prisoner's Dilemma Maximizing-Difference game (IPD-MD; Halevy et al., 2008), they find that subjects opt to benefit their in-group at a cost to a defenseless out-group even when they could achieve the same end without harming that out-group (see, e.g., Böhm et al., 2016, for complementary findings).

Also using variants of the IPD-MD, Weisel finds that subjects are largely consistent in their parochially altruistic choices when they decide (a) to harm or (b) not to help an out-group. Interestingly, though, Weisel also finds that subjects are reluctant to harm out-groups whom they have had the possibility to help before. This is, thus, first evidence for order effects in parochially altruistic choice.

De Dreu et al. investigate the interaction of deliberate reasoning and parochial altruism. Extending earlier findings on intuitive cooperativeness in dyadic settings (Rand et al., 2012; Peysakhovich et al., 2014) to the intergroup context, they find evidence of increased parochialism in the IPD-MD when subjects were cognitively taxed. Their seminal findings thus suggest that parochially altruistic choice might operate through intuitive mechanisms.

In a similar vein, Reimers and Diekhof closely investigate potential mechanisms coupling in-group cooperation and defection against out-groups. In their study employing dyadic Prisoner's Dilemma games (PDGs) played by male subjects belonging either to the same or to different natural groups, they find that testosterone levels positively correlate with revealed in-group favoritism.

Dorrough et al. employ repeated dyadic PDGs to study the dynamic development of parochially altruistic choice over time. While not showing an initial difference in cooperation levels between PDGs played with either in- or out-group members, subjects in this study gradually formed more positive expectations about their in-group members' cooperative behavior, eventually leading to pronounced in-group favoritism.

In two field experiments employing the lost-letter paradigm, Hellmann et al. find that reluctance to help members of stigmatized out-groups is conditional on the respective out-group members' social status and that an in-group member trying to contact an out-group member is more likely to be helped than an out-group member trying to contact another out-group member.

While the aforementioned studies investigate parochial altruism at the individual level, Wildschut et al. and Frischlich et al. take a closer look at how parochially altruistic norms may be formed and disseminated at the group level. Employing dyadic PDGs, Wildschut et al. find that increased normative group pressure induced by introducing accountability of individual PDG choices amplifies the inter-individual inter-group discontinuity effect (Wildschut et al., 2003). Frischlich et al. study the reaction of subjects to parochially altruistic norms conveyed in extremist propaganda videos and find that subjects submitted to a mortality salience prime report a higher level of interest in such propaganda.

In addition to these findings that shed fresh light on the antecedents of parochially altruistic choice, two contributions present negative results, highlighting the importance of refined theorizing.

In an elaborate field study conducted in Northern Ireland before, during, and after an outbreak of violent intergroup conflict, Silva and Mace find that charitable giving to neutral and out-group but, remarkably, also to in-group institutions was significantly reduced during the time of conflict, questioning simple notions of an unconditional link of conflict levels to increases in “in-group love.”

Corr et al. finally, use dyadic Trust Games and PDGs to investigate the association of general pro-sociality and in-group favoritism within individuals. Strikingly, they find that pro-sociality does not predict in-group favoritism in these games. Furthermore, they even find that these two traits are predicted by separate Big-5 personality dimensions.

In summary, we are positive that the instructive evidence gathered here will inspire refined work on parochial altruism. This Research Topic, we hold, marks a fruitful starting point for exciting progression.

## Author contributions

All authors listed, have made substantial, direct and intellectual contribution to the work, and approved it for publication.

### Conflict of interest statement

The authors declare that the research was conducted in the absence of any commercial or financial relationships that could be construed as a potential conflict of interest.
